# Novel ceRNA network construction associated with programmed cell death in acute rejection of heart allograft in mice

**DOI:** 10.3389/fimmu.2023.1184409

**Published:** 2023-09-11

**Authors:** Yiwen Guo, Yixi Zhang, Jia Yu, Yuqi Dong, Zhitao Chen, Chuchen Zhu, Xitao Hong, Zhonghao Xie, Min Zhang, Shuai Wang, Yichen Liang, Xiaoshun He, Weiqiang Ju, Maogen Chen

**Affiliations:** ^1^ The First Affiliated Hospital, Sun Yat-Sen University, Organ Transplant Centre, Guangzhou, China; ^2^ Guangdong Provincial Key Laboratory of Organ Donation and Transplant Immunology, Guangzhou, China; ^3^ Guangdong Provincial International Cooperation Base of Science and Technology (Organ Transplantation), Guangzhou, China; ^4^ Liver Transplantation Center, Beijing Friendship Hospital, Capital Medical University, Beijing, China

**Keywords:** ceRNA, heart transplant, acute rejection (AR), lncRNA, microRNA, mRNA

## Abstract

**Background:**

T cell-mediated acute rejection(AR) after heart transplantation(HT) ultimately results in graft failure and is a common indication for secondary transplantation. It’s a serious threat to heart transplant recipients. This study aimed to explore the novel lncRNA-miRNA-mRNA networks that contributed to AR in a mouse heart transplantation model.

**Methods:**

The donor heart from Babl/C mice was transplanted to C57BL/6 mice with heterotopic implantation to the abdominal cavity. The control group was syngeneic heart transplantation with the same kind of mice donor. The whole-transcriptome sequencing was performed to obtain differentially expressed mRNAs (DEmRNAs), miRNAs (DEmiRNAs) and lncRNAs (DElncRNAs) in mouse heart allograft. The biological functions of ceRNA networks was analyzed by GO and KEGG enrichment. Differentially expressed ceRNA involved in programmed cell death were further verified with qRT-PCR testing.

**Results:**

Lots of DEmRNAs, DEmiRNAs and DElncRNAs were identified in acute rejection and control after heart transplantation, including up-regulated 4754 DEmRNAs, 1634 DElncRNAs, 182 DEmiRNAs, and down-regulated 4365 DEmRNAs, 1761 DElncRNAs, 132 DEmiRNAs. Based on the ceRNA theory, lncRNA-miRNA-mRNA regulatory networks were constructed in allograft acute rejection response. The functional enrichment analysis indicate that the down-regulated mRNAs are mainly involved in cardiac muscle cell contraction, potassium channel activity, etc. and the up-regulated mRNAs are mainly involved in T cell differentiation and mononuclear cell migration, etc. The KEGG pathway enrichment analysis showed that the down-regulated DEmRNAs were mainly enriched in adrenergic signaling, axon guidance, calcium signaling pathway, etc. The up-regulated DEmRNAs were enriched in the adhesion function, chemokine signaling pathway, apoptosis, etc. Four lncRNA-mediated ceRNA regulatory pathways, Pvt1/miR-30c-5p/Pdgfc, 1700071M16Rik/miR-145a-3p/Pdgfc, 1700071M16Rik/miR-145a-3p/Tox, 1700071M16Rik/miR-145a-3p/Themis2, were finally validated. In addition, increased expression of PVT1, 1700071M16Rik, Tox and Themis2 may be considered as potential diagnostic gene biomarkers in AR.

**Conclusion:**

We speculated that Pvt1/miR-30c-5p/Pdgfc, 1700071M16Rik/miR-145a-3p/Pdgfc, 1700071M16Rik/miR-145a-3p/Tox and 1700071M16Rik/miR-145a-3p/Themis2 interaction pairs may serve as potential biomarkers in AR after HT.

## Background

Heart transplantation is an effective method of treating patients with end-stage heart failure. However, patients are at risk for several complications after transplantation, the most common of which is acute rejection. Recipients mortality due to acute rejection has been reported up to 11% within 3 years after heart transplantation (1).

Over the past decade, with the appearance of numerous high-throughput genomic platforms and bioinformatics, it has been found that more than 90% of genes in the genome belong to non-coding RNA (non-coding RNA, ncRNA), and less than 2% of genes belong to proteins-encoding RNA (2–4). long non-coding RNA(lncRNA), which comprises a variety of RNA species longer than 200nt, is the majority of non-coding RNA in human body (5). MiRNA is a single-stranded RNA of 21-25 nt in length that binds to other RNAs through complementary nucleotide sequences to influence the function and translation of other RNAs and then regulate gene expression.

In heart transplantation, ncRNAs are involved in the process of heart transplant rejection mainly by regulating immune and inflammatory responses. LncRNAs can modulate the immune response and affect graft survival. Studies have reported that lncRNAs alter the recipient immune environment by regulating Treg, Th1 or DC cell ratios or phenotypic alterations, affecting immune rejection and regulating graft survival (6–8). MiRNAs are also closely related to transplantation immune rejection. miRNAs regulate the immune and inflammatory microenvironment in the recipient, and induce immune tolerance or rejection in the graft (9–13). Therefore, differential expression of miRNAs can be used to predict rejection after heart transplantation (14–19). It is worth mentioning that although miRNA has been shown to predict heart transplant rejection, some studies have demonstrated that the predictive power of miRNA is still far from adequate compared to troponin T (20). Besides, circle RNA can also be involved in immune rejection of heart transplantation (21).

In this study, we used whole transcriptome sequencing technology to preliminarily explore and validate the ceRNA regulatory network affecting acute rejection and to elucidate the possible biomarkers of acute rejection. Here, we tentatively propose RNAs critical for acute rejection of heart transplantation that may play a role in various immune responses after heart transplantation.

## Methods

### Animals

Babl/C and C57/B6 mice (6-8 weeks) were purchased from Guangdong Medical Laboratory Animal Center. The mice were housed in the animal facility of Sun Yat-sen University, where they were kept in a specific pathogen-free environment with a 12:12-hour light-dark cycle, on sterile chow and sterilized water. All animal experiments were approved by the ethics committee of the First Affiliated Hospital, Sun Yat-sen University.

### Heart transplantation and histology

We construct the mice heart transplantation acute rejection model by transplanting the heart of Babl/c or C57BL/6 donor mice into the abdominal cavity of C57/B6 mice, as previously reported (22). Allogeneic heart transplantation was defined as the acute rejection(AR) group, and homogeneous heart transplantation was the control(CON) group. After transplantation, the rejection of the transplanted heart was determined by observing the abdominal pulsation. Rejection was considered to occur if the heartbeat stopped. Specimens of transplanted hearts were embedded and sections, stained with hematoxylin/eosin for microscopic evaluation.

### Screening strategy for DEmRNAs, DElncRNAs and DEmiRNAs

The differential expression of mRNAs, lncRNAs and miRNAs between the AR and CON groups was analyzed using the DESeq2 method. The screening thresholds for significant differences in gene expression were adjusted to p<0.05 and |log2FC(fold change)|>1.5. Heat maps and volcano maps were generated for visual analysis.

### lncRNA-miRNA-mRNA regulatory network

The DElncRNA-DEmiRNA-DEmRNA network was constructed based on the ceRNA hypothesis: (1) the interaction information of miRNA-mRNAs in miRmap, Miranda, miRDB, TargetScan and MitarBase and miRNA-lncRNAs in Starbase was extracted; (2) If both lncRNAs and mRNAs were targeted and negatively expressed with a common miRNA, the lncRNA-miRNA-mRNA set was identified as a co-expression competition triad and the corresponding ceRNA regulatory network was constructed. ceRNA regulatory network was visualized with Cytoscape 3.7.1

### KEGG and GO function enrichment analysis

To predict the potential biological functions of genes in the ceRNA network, the GO pathway (e.g., biological process, BP; cellular component, CC and molecular function, MF) and the KEGG pathway were implemented using the ClueGO app. p<0.05 was considered statistically significant and the results were visualized with bubble plots.

### PCR

Total RNA of the heart was extracted using RNA Extraction Kit (Accurate Biology, Hunan, China) following the manufacturer’s instructions. The Evo M-MLV RT Premix for qPCR (Accurate Biology, Hunan, China) and a polymerase chain reaction (PCR) System generated cDNA. The PCR was performed with the SYBR Green Premix Pro Taq HS qPCR KIT (Accurate Biology, Hunan, China). The results were analyzed by the 2^-ΔΔCT^ method. Gene expression data were shown as relative to the control group, which was set as 100%.

### Statistical analysis

In this study, GraphPad Prism 8 (GraphPad Software, San Diego, California, USA) was used for data statistics and analysis. Differences in the expression of mRNAs, lncRNAs and miRNAs between the AR group and the CON group were analyzed using t-test. p<0.05 was considered a significant difference.

## Results

### Construction of acute rejection model of heart transplantation in mice

**Figure 1:**
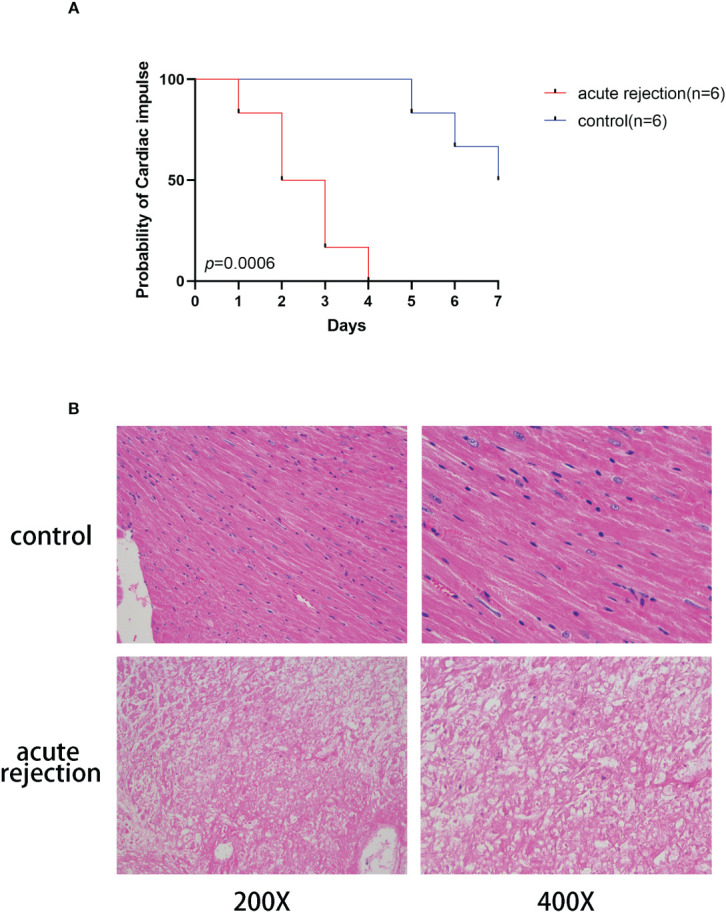
Construction of acute rejection model of heart transplantation in mice. **(A)**The cardiac arrest curve in mice within 7 days after transplantation. Red represents the acute rejection group(n=6) and blue indicates the control group(n=6). **(B)** Myocardial pathology in control and acute rejection group.

The mice acute rejection model was constructed by allogeneic heart transplantation, and cardiac allograft rejection or survival was confirmed by visualization and unpalpation. We constructed Allogeneic and homogeneous heart transplantation for comparative analysis. The survival of graft was shown in **Figure 1A**, the AR group (n=6) started to show rejection on day 1 after transplantation, and all grafts stopped beating by day 4. While the CON group (n=6) began to show rejection on day 6 and by the end of the observation period three grafts stopped beating, which was a significant difference. The mice transplanted hearts were subsequently subjected to pathological analysis. The transplanted hearts in the AR group showed a series of acute rejection manifestations such as myocardial vacuolar lesions, disorganized cell morphology, and loss of nuclei, while the hearts from the CON group showed normal cardiomyocyte manifestations, as shown in **Figure 1B**.

### Screening of DEmRNAs, DElncRNAs and DEmiRNAs

**Figure 2:**
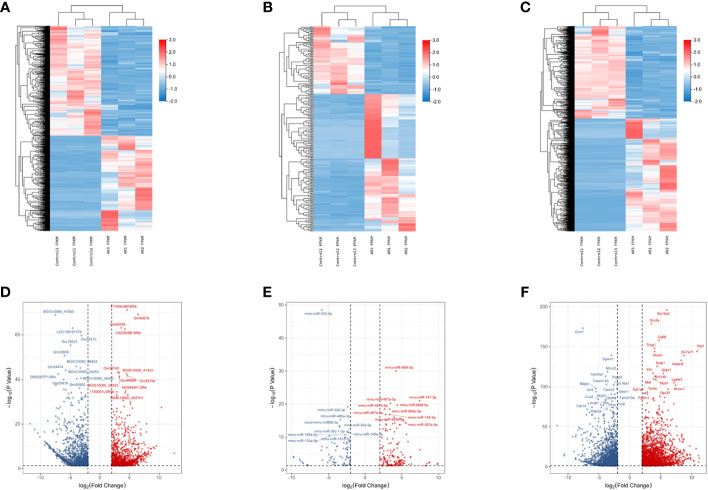
Heatmap plots and volcano plots of DelncRNAs, DemiRNAs, and DemRNAs during rejection and control group in an acute rejection heart transplant model. A comparative analysis for expression profiles of lncRNAs, miRNAs and mRNAs between allogeneic transplantation group and syngenic transplantation controls was performed with P < 0.05 and |log2 fold change [FC]|>1.5 as threshold. **(A)** Heatmap of differentially expressed lncRNAs; **(B)** Heatmap of differentially expressed miRNAs; **(C)** Heatmap of differentially expressed mRNAs; **(D)** Volcano plot of 2175 differentially expressed lncRNAs; **(E)** Volcano plot of 314 differentially expressed miRNAs; **(F)** Volcano plot of 3958 differentially expressed mRNAs. Red represents upregulated genes and blue indicates downregulated genes. DE, differentially expressed; FC, fold change.

The whole-transcriptome sequencing results demonstrated 12925 DE-RNAs significantly associated with acute rejection. There were 9119 DE-mRNAs in the transcriptome, including 4754 up-regulated DEmRNAs and 4365 down-regulated DEmRNAs. Likewise, there were 1634 up-regulated DE-lncRNAs, 1761 down-regulated DE-lncRNAs, 182 up-regulated DE-miRNAs and 132 down-regulated DE-miRNAs. The heat map and volcano plot of DEmRNAs, DElncRNAs and DEmiRNAs were shown in **Figure 2**.

### Differential gene interaction network construction

**Figure 3:**
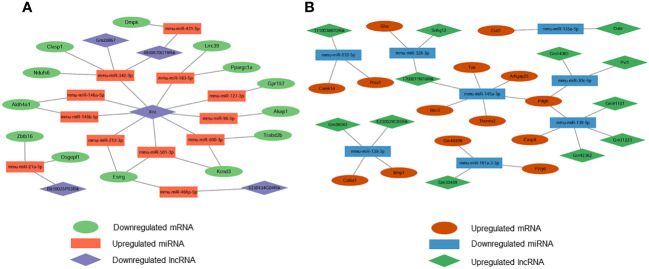
Construction of lncRNA-mediated ceRNA regulatory network. **(A)** The lncRNA-mediated downregulated ceRNA network; **(B)** The lncRNA-mediated upregulated ceRNA network. Green means downregulated LncRNA, red means upregulated LncRNA, blue indicates upregulated miRNAs and mRNAs, yellow represents downregulated miRNAs, orange represents downregulated mRNAs.

We constructed DE-miRNA-mediated ceRNA regulatory networks based on base sequences and expression levels. Based on the interaction elements, 14 miRNA-lncRNA pairs and 16 miRNA-mRNA pairs were identified in the upregulated miRNA ceRNA network, and 13 miRNA-lncRNA pairs and 16 miRNA-mRNA pairs in the downregulated miRNA ceRNA network. Based on this, the down-regulated ceRNA network including 5 lncRNA nodes, 12 miRNA nodes and 13 mRNA nodes, and the up-regulated ceRNA network including 12 lncRNA nodes, 8 miRNA nodes and 14 mRNA nodes were constructed. The downregulated and upregulated ceRNA networks were shown in **Figure 3** separately.

### KEGG and GO functional enrichment analysis

**Figure 4:**
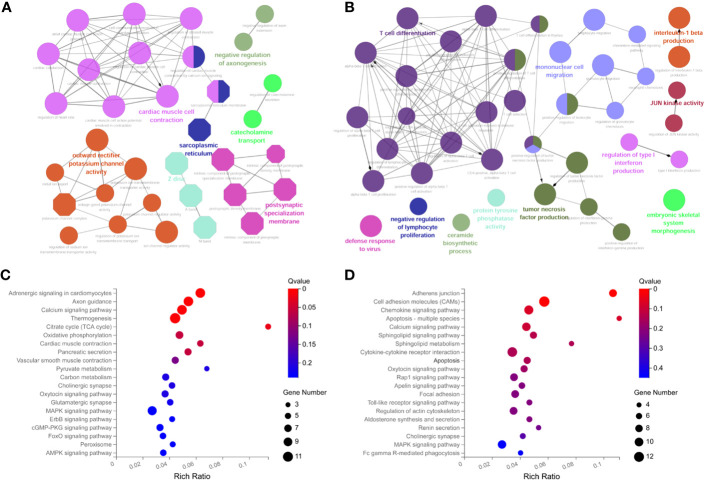
Functional enrichment analysis of DemRNAs in ceRNA networks with ClueGO app. **(A)** GO biological functional analyses of downregulated mRNA; **(B)** GO biological function analyses of upregulated mRNA; **(C)** KEGG pathway analyses of downregulated mRNA; **(D)** KEGG pathway analyses of upregulated mRNA. BP, biological process; CC, cellular component; MF, molecular function; GO, Gene Ontology.

To further explore the potential functions of ceRNA network-related RNAs, we performed functional enrichment analysis using the ClueGO app. The results indicate that the down-regulated mRNAs are mainly involved in cardiac muscle cell contraction, potassium channel activity, etc (**Figure 4A**). the up-regulated mRNAs are mainly involved in T cell differentiation and mononuclear cell migration, etc (**Figure 4B**). The KEGG pathway enrichment analysis showed that the down-regulated DEmRNAs were mainly enriched in adrenergic signaling, axon guidance, calcium signaling pathway, etc (**Figure 4C**). The up-regulated DEmRNAs were enriched in the adhesion function, chemokine signaling pathway, apoptosis, etc (**Figure 4D**).

### qRT-PCR validation

**Figure 5:**
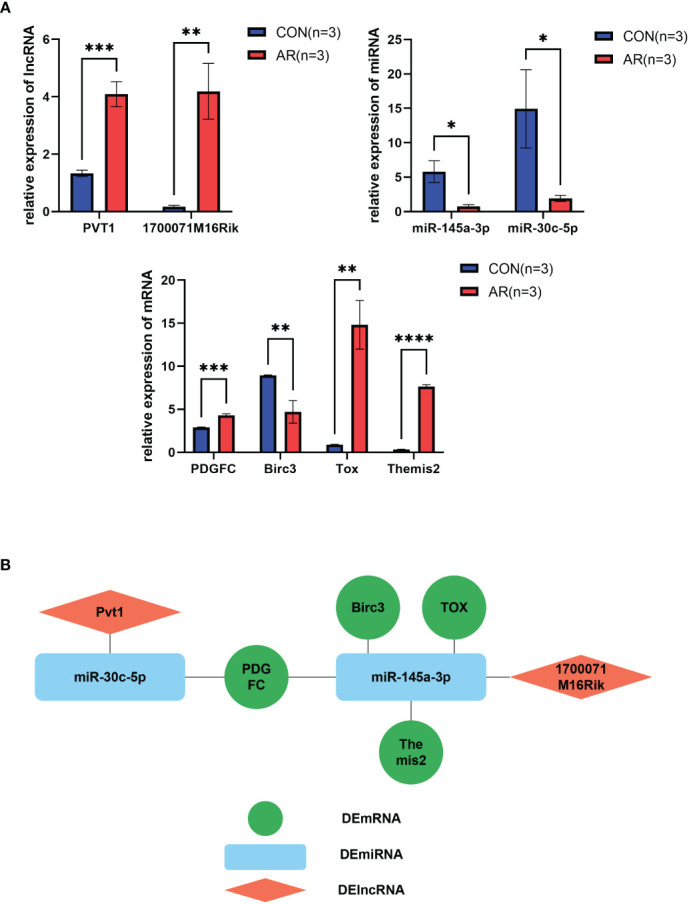
Validation of key RNAs in ceRNA networks. **(A)** Significance qPCR validation results of DEmiRNA, DElncRNA and DEmRNA within ceRNA networks(n=3). **(B)** Diagram of key RNA regulatory networks. Diamond shape represents DElncRNA, rounded rectangle represents DEmiRNA, circle represents DEmRNA. *P<0.05, **P<0.01, ***P<0.001, ****P<0.0001.

To find the key genes in the ceRNA network, we validated the DERNAs found by sequencing *in vivo* (n=3) by qRT-PCR, and the results were shown in **Figure 5A**. We identified genes that play a key role in acute rejection, including PDGFC(*t*=11.200, *p*<0.000), Birc3(*t*=3.829,*p*<0.001), TOX(t=13.770, *p*<0.000), Themis2(*t*=23.770, *p*<0.000), PVT1(*t*=11.420, *p*<0.000), 1700071M16Rik(*t*=8.672, *p*<0.000), miR-145a-3p(*t*=10.530,*p*<0.000), miR-30c-5p(*t*=7.901, *p*<0.000). The interaction network was shown in **Figure 5B**.

### Identification of programmed cell death-associated genes in ceRNA network

We compared 75 differentially expressed mRNAs in the ceRNA network with programmed cell death-related genes in GeneCard and obtained 62 common genes. Comparing these common genes with the key genes validated above, we found that among them pyroptosis gene Birc3, and apoptosis genes PDGFC, TOX and THEMIS2 were common, and these genes are likely to be involved in cell death caused by acute rejection.

## Conclusion

In our study, we confirmed the existence of lncRNA-miRNA-mRNA interactions network in the heart transplantation acute rejection model. The regulatory network between mRNA, lncRNA and miRNA was constructed based on the ceRNA network theory by comparing the whole transcriptome of acute rejection group and control group. KEGG and GO analyses demonstrated that genes within this network are mainly involved in inflammation and immune-related functions. Finally, qRT-PCR validated the key genes in this network, including Pvt1/miR-30c-5p/Pdgfc, 1700071M16RIK/miR-145a-3p/Pdgfc, 1700071M16RIK/miR-145a-3p/TOX, 1700071M16RIK/miR-145a-3p/THEMIS2.

To our knowledge, PDGFC and PVT1 have been shown to be associated with acute rejection of transplanted hearts. PDGFC, a member of platelet-derived growth factor (PDGF), has been reported to be associated with chronic rejection of cardiac transplantation, upregulating TGF-β1 and promoting myocardial fibrosis and atherosclerosis (23). Overexpression of PVT1 upregulates TNF receptor-associated factor (TRAF) 6 expression through targeting miRNAs to alter Treg autophagy and inhibit cardiac transplant rejection (6).

Themis2 is associated with B-cell development and macrophage immune responses (24, 25).TOX regulates transcriptional processes by binding to DNA in a structure-dependent way. TOX is involved in T cell development and autoimmune regulation, and plays a critical role in T cell depletion (26). BIRC3 is one of the eight members of the human inhibitors of apoptosis proteins family. The literature reports that Birc3 expression is associated with tumor prognosis, inflammatory response and immune disorders (27–29). Birc3 inhibits cellular pyroptosis by inactivating NLRC4 inflammatory vesicles (30).

miR-30c-5p has been shown to be associated with cardiac IRI and ameliorates myocardial injury by suppressing apoptosis-related genes such as BCL2, Bach1 (31, 32). Chen J et al. reported that miR-30c-5p can activate NF-κB pathway to promote myocardial IRI (33). miR-145a-3p is associated with lipid metabolism, but the relationship with rejection is unclear (34). Besides, miR-30c-5p may be associated with immunosuppression/graft tolerance induction in liver transplant recipients after transplantation. Morsiani C et al. found that miR-30c-5p decreased in liver transplant patients in early to mid-term follow-up, but returned to normal levels 19 months after liver transplantation (35).

Antibody-mediated rejection is a major cause of heart transplant failure, and previous studies have reported that C4d deposition is associated with heart transplants acute rejection (36, 37). Using C4d-targeted microbubbles loaded with nitric oxide could improve the therapeutic efficacy of heart transplant rejection (38). We found that the positivity of C4d was higher in the AR group compared to the CON group (p=0.0159), the results are shown in **Supplementary Figure 1**. The relationship between C4d deposition and the above RNAs has not yet been reported, and it remains to be investigated which kind of RNA could monitor the C4d deposition.

In this study, we propose the ceRNA regulatory network in heart transplantation acute rejection and elucidate the possible mechanisms underlying the influence of this regulatory network on the survival of transplanted hearts. However, this study only used a mice heart transplantation model and was not validated *in vitro* or patients, so the ceRNA network may not apply to humans. In the future, we will continue to conduct *in vitro* experiments and collect serum from patients for further analysis. In addition, this study has not verified the protein expression alteration of differential mRNAs, and since the function of mRNAs needs to be further exerted at the protein level, the regulatory network may also be altered due to translation modifications.

## Data availability statement

The datasets presented in this study can be found in online repositories. The names of the repository/repositories and accession number(s) can be found below: https://www.ncbi.nlm.nih.gov/, PRJNA943392.

## Ethics statement

The animal study was approved by Medical Ethics Committee, The First Affiliated Hospital of Sun Yat-sen University. The study was conducted in accordance with the local legislation and institutional requirements.

## Author contributions

YG, MC and YZ contributed to conception and design of the study. YD and ZC established the animal model. XtH, MZ and ZX performed the statistical analysis. SW and YL performed the bioinformatic analysis. WJ and XsH wrote the first draft of the manuscript. CZ, YG and YZ wrote sections of the manuscript. All authors contributed to the article and approved the submitted version.
