# Supplementary figures and images for: Novel ceRNA network construction associated with programmed cell death in acute rejection of heart allograft in mice
Source: Front Immunol. 2023 Sep 11;14:1184409. doi: 10.3389/fimmu.2023.1184409 (PMC10518384; doi:10.3389/fimmu.2023.1184409)

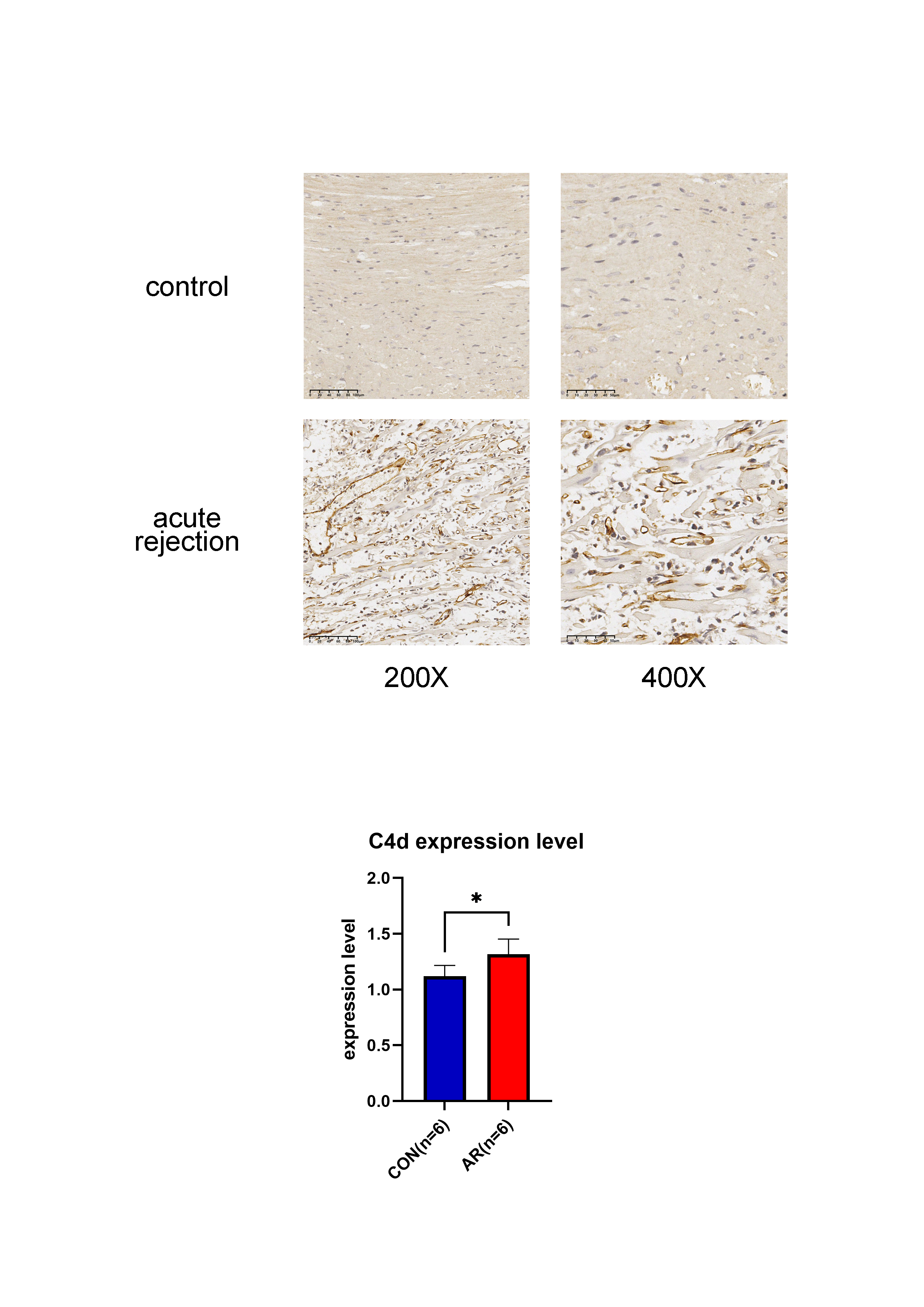

Supplement: Supplementary file 1 [file Image_1.tif]
